# Peptides from Tetraspanin CD9 Are Potent Inhibitors of *Staphylococcus Aureus* Adherence to Keratinocytes

**DOI:** 10.1371/journal.pone.0160387

**Published:** 2016-07-28

**Authors:** Jennifer K. Ventress, Lynda J. Partridge, Robert C. Read, Daniel Cozens, Sheila MacNeil, Peter N. Monk

**Affiliations:** 1 Department of Infection, Immunity and Cardiovascular Diseases, University of Sheffield Medical School, Sheffield, United Kingdom; 2 Department of Molecular Biology and Biotechnology, University of Sheffield, Sheffield, United Kingdom; 3 Clinical and Experimental Medicine, University of Southampton, Southampton, United Kingdom; 4 Materials Science and Engineering, University of Sheffield, Sheffield, United Kingdom; University Hospital Hamburg-Eppendorf, GERMANY

## Abstract

*Staphylococcus aureus* is one of the primary causative agents of skin and wound infections. As bacterial adherence is essential for infection, blocking this step can reduce invasion of host tissues by pathogens. An anti-adhesion therapy, based on a host membrane protein family, the tetraspanins, has been developed that can inhibit the adhesion of *S*. *aureus* to human cells. Synthetic peptides derived from a keratinocyte-expressed tetraspanin, CD9, were tested for anti-adhesive properties and at low nanomolar concentrations were shown to inhibit bacterial adhesion to cultured keratinocytes and to be effective in a tissue engineered model of human skin infection. These potential therapeutics had no effect on keratinocyte viability, migration or proliferation, indicating that they could be a valuable addition to current treatments for skin infection.

## Introduction

Bacterial skin infections are characterised by an uncontrolled and excessive growth of bacterial pathogens in or on the skin, and are an important healthcare challenge. Infections begin commonly in wounds and burns, and in patients with psoriasis or other skin conditions in which the natural skin barrier is damaged. *Staphylococcus aureus* is a common causative pathogen in skin and wound infections and once established in the skin causes noticeable symptoms including dryness, pruritus, and pain, as well as clinical conditions such as cellulitis, folliculitis, furuncles and impetigo, and potentially fatal systemic infection [1].

Like most bacterial skin pathogens, *S*. *aureus* has to gain access to target tissues via a break in the stratum corneum and then attach to underlying cells to cause an infection. *S*. *aureus* has a range of adhesins that allow it to adhere tightly to molecules associated with the host cell surface. Such host receptor molecules include the extracellular matrix protein fibronectin, scavenger receptors such as CD36 and surface-expressed chaperone Hsc70. All of these adhesion target molecule are known to interact with a family of eukaryotic membrane proteins known as tetraspanins, which act as molecular facilitators [2]. Tetraspanins are membrane proteins characterized by 4 transmembrane domains, containing charged residues, 1 intracellular loop, 2 intracellular termini and 2 extracellular loops, the second of which (EC2 domain) makes specific protein-protein interactions. Tetraspanins associate with each other in the membrane via membrane-proximal palmitoylation sites, as well as associating with other cell components such as signalling molecules, structural proteins and G-protein coupled receptors, in order to form tetraspanin-enriched microdomains (TEM). TEM have been implicated in many cell functions, including cell adherence and fusion, membrane trafficking, endocytosis, leukocyte adherence and motility but can also be exploited by protozoa, viruses and bacteria as gateways for infection [3, 4]. For example, uropathogenic *E*. *coli* have been shown to exploit tetraspanins in order to adhere to bladder cells through the *E*. *coli* adhesin, FimH, binding directly to tetraspanin TSPAN21 [5]. More commonly, bacterial adhesion requires an indirect interaction with tetraspanins, through receptors embedded in TEM [6]. Thus tetraspanins are likely to make useful targets for novel anti-infectives, particularly if TEM function can be disrupted; this is likely to result in the disorganisation of multiple potential bacterial receptor proteins and also affect the binding of multiple species of bacteria.

Previously we have shown that the application of anti-tetraspanin antibodies or recombinant EC2 domains of some tetraspanins can disrupt TEM on endothelial cells, resulting in the disorganisation of integrins and decreased adhesion of lymphocytes under flow conditions in vitro and in vivo [7, 8]. More recently, we also found that the recombinant EC2 domain of CD9 but not the closely related tetraspanin CD81, could substantially decrease the adherence of multiple species of bacteria such as *Neisseria meningitidis* and *Salmonella enterica* to mammalian cells [9]. Here we show that short (14/15 amino acid) peptides directly derived from the sequence of the EC2 domain of CD9 have potent anti-adhesive effects against *Staphylococcus aureus* in epithelial cell lines, primary keratinocytes and, importantly, in a 3D tissue-engineered model of human skin. We also show that these peptides have no adverse effects on cell metabolism or epidermal migration, indicating that this may be an important new class of anti-bacterial agents.

## Materials and Methods

### Ethics Statement

All work using human keratinocytes and fibroblasts was performed on samples from abdominoplasty and breast reduction. Participants provided their written informed consent to donate the skin, which was stored in the patient’s clinical notes. The protocol and consent form were approved by the local ethics committee Sheffield NHS Trust, Sheffield, UK. Tissues and cells were stored and used on an anonymous basis under UK Human Tissue Authority Research Tissue Bank Licence Number 12179.

### Bacterial Strains

SH1000 is a laboratory strain provided by Simon Foster (University of Sheffield, UK), expressing a chloramphenicol resistance plasmid (pSK5487) with a gfp gene. MRSA strain JE2 is derived from USA300, a well characterised clinical isolate of community acquired multi-drug resistant *Staphylococcus aureus*. It was produced by removing a macrolide resistance plasmid and a cryptic plasmid and was kindly provided by Andrew Liew Tze Fui (University of Technology, Sydney, Australia). S235 is a virulent clinical isolate of Community Associated-MRSA (CA-MRSA) kindly donated by the Sheffield School of Clinical Dentistry. Bacteria were routinely grown in Brain Heart Infusion Media (Oxoid, Basingstoke, UK), and bacteria were grown in log phase (as determined by both optical density at 600nm (OD_600_) and CFU counts) for infection assays.

### Cell Lines and Primary Skin Cells

HaCaT cells are a human keratinocyte based cell line obtained from Cell Line Services (CLS GmbH, Eppelheim, Germany), routinely cultured in DMEM (Sigma-Aldrich, Gillingham, UK) plus 10% foetal calf serum (FCS, Bioscience, UK). Normal primary keratinocytes and fibroblasts were cultured from skin donated from patients having abdominoplasty and breast reduction surgeries. Samples were processed to isolate normal human epidermal keratinocytes and dermal fibroblasts, and remaining skin salt split with 1M NaCl to de-epidermise and decellularise using a protocol described in Shepherd et al [10] forming a dermal scaffold for cell seeding. Keratinocytes were cultured either in Green’s medium (produced in-house, as described in Little et al. [11]) to produce a differentiated state or MCDB153 (Sigma-Aldrich, Gillingham, UK), a low calcium, low protein media that inhibits keratinocyte differentiation [11]. Dermal fibroblasts were cultured in DMEM media with 10% FCS.

### Peptides

**Fig 1:**
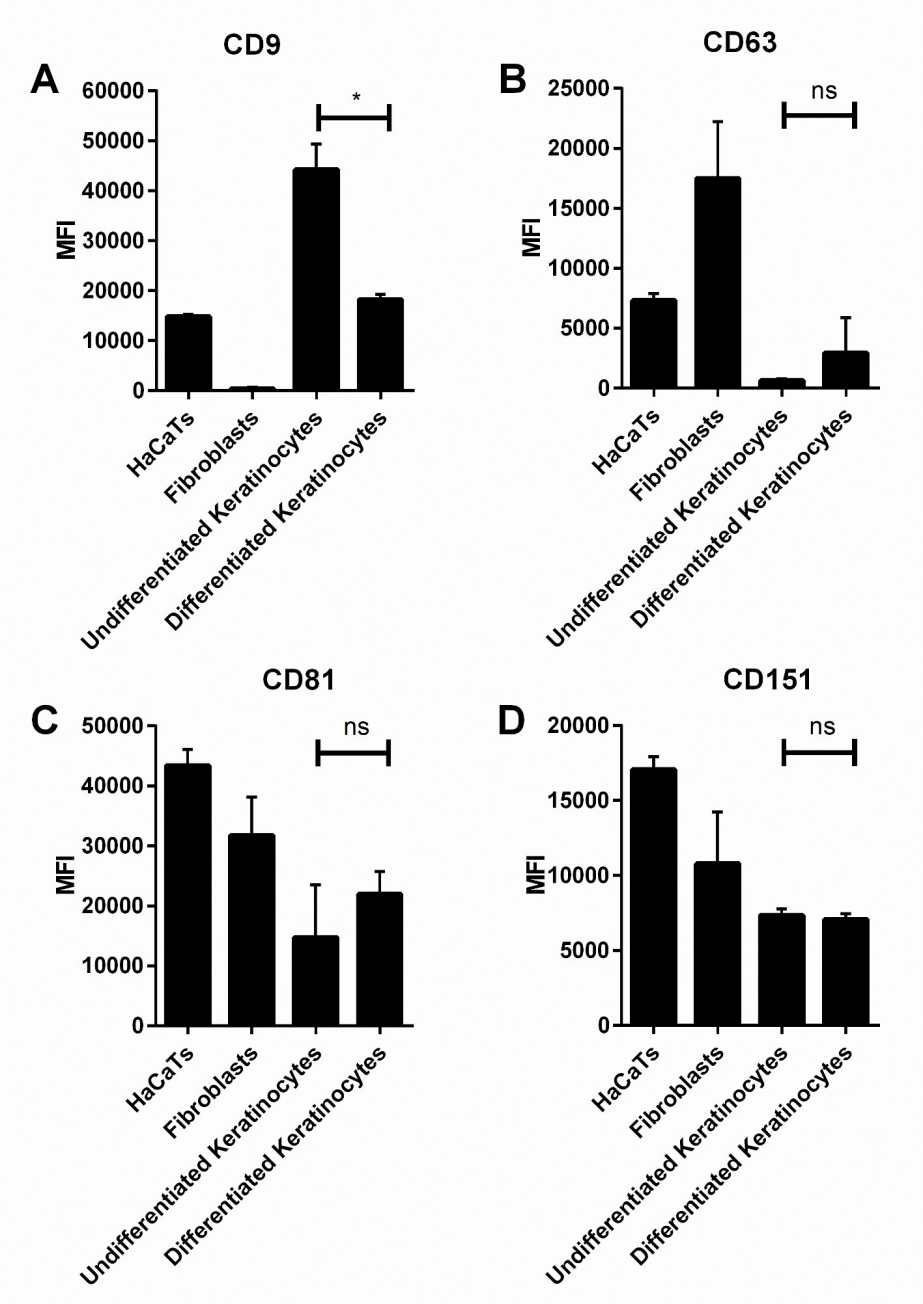
Tetraspanin Expression on Skin Cells. The expression levels of CD9, CD63, CD81 and CD151 on HaCaT cells, fibroblasts and keratinocytes were quantified by flow cytometry. The expression of each tetraspanin was compared for differentiated primary keratinocytes grown in Green’s medium and undifferentiated keratinocytes from the same donor grown in MCDB15132 low calcium medium. Data are from 3 separate HaCaT cultures, 10 individual fibroblast donors and 2 individual keratinocyte donors. * p≤0.05, unpaired t test.

The EC2 region of CD9 (Fig 1A) was divided into 14–15 residue segments, which were synthesized using solid phase Fmoc chemistry (Genscript, New Jersey, USA). Scrambled peptides were randomly generated from the cognate CD9 sequence.

### Cell Line Infection

Cells were seeded onto 12 mm diameter glass coverslips within 24-well cell culture plates in the following quantities, then incubated overnight at 37°C with 5% CO_2_ before use: normal human keratinocytes at 3x10^5^ cells per well, dermal fibroblasts at 7.5x10^4^, and HaCaT cells at 3.75x10^4^. After overnight culture wells were washed with PBS and blocked with 5% BSA (FirstLink, Wolverhampton, UK). The wells were then washed again with PBS and incubated for 30 minutes with 50nM peptide before infection with *Staphylococcus aureus* for 1 hour. Multiplicities of infection varied between cell types, but were determined in preliminary experiments to give an infection rate of approximately 20–40% of cells. After washing 4 times in PBS, the infected cells were then fixed with 2% paraformaldehyde, stained with 0.5μg ml^-1^ of 4, 6-diamidino-2-phenyl-indole hydrochloride (DAPI, Molecular Probes, Eugene, USA) and quantified using fluorescent microscopy. Each coverslip was analysed by a random count of 100 cells, scoring for the number of cells with bacteria attached, and number of attached bacteria per cell. Cells undergoing mitosis were considered abnormal and therefore not scored. Maximal peptide efficacy was calculated as the net rate of inhibition of adhesion (peptide-scrambled control) of SH1000 bacteria by the most effective peptide in each case using the ‘percentage of cells with adherent bacteria’ measure, the raw data for which is shown in S1 Fig.

### Cell Viability

MTT (3-(4,5-dimethylthiazol-2-yl)-2,5-diphenyltetrazolium bromide) was used to assess cell viability. HaCaT cells were grown in a 24 well plate for 24 hours, then treated for a further 24 hours with 200nM peptides in media. Peptide was then removed and the tetrazolian MTT dye (Sigma-Aldrich, Poole, UK) added at a concentration of 0.5mg/ml in cell media for 1 hour. Cells were then lysed with 2-butoxyethanol (Sigma-Aldrich, Poole, UK) and the absorbance of supernatants measured at 562nm.

### Tissue Engineered Skin (TEskin) Wound Infection Model

Small pieces of de-epidermised dermis (DED) were placed in 12 well culture plate inserts with 0.4μm pores (ThinCert, Griener Bio-One GmbH, Germany) to allow media diffusion. Keratinocytes (between passages 1 and 2) were seeded on top of the DED at a density of 500,000 cells per composite, and fibroblasts were seeded at a density of 150,000 cells per composite. The surrounding wells were filled with Greens media plus 10% FCS and the TEskin cultured for 3 days at 37^°^C 5% CO_2_ in submerged culture. At day 3, media was replaced, and the TEskin cultured at an air-liquid interface for 10–14 days. On the day of infection the TEskin was wounded by applying a 4mm rod heated in a Bunsen flame to each TEskin piece for 3 seconds. The TEskin pieces were then washed three times with PBS before the treatment was applied. 200nM of the relevant peptide and scrambled control were applied to the TEskin in 100μl serum-free DMEM and serum-free DMEM without additions was added to the remaining untreated TEskin. A fresh colony of *S*. *aureus* strain S235 was grown to exponential growth phase, harvested and washed three times with PBS and bacterial number estimated by the OD_600_nm. The bacteria were then re-suspended at a concentration of 1x10^8^/ml according to a standardized growth curve and 100μl of this was then applied to the TEskin 1 hour after wounding/treatment. The sample was incubated for 5 further hours at 37°C in a humidified environment of 5% CO_2_, washed to remove non-adherent bacteria, and left for a further 18 hours before bacterial isolation. TEskin was washed, cut in half, weighed and treated with 2mg/ml saponin (Sigma-Aldrich, UK) for 12 min before diluting and plating to obtain viable counts. Saponin at this concentration had no effect on bacterial growth or viability, even after 24 h in culture.

### Epidermal and Cell Migration Assays

Epidermal migration was assessed by seeding 3x10^5^ keratinocytes and 1x10^5^ fibroblasts into the centre of a 10mm diameter metal ring on top of a de-cellularised dermal scaffold and allowing the cells to grow submerged for 3 days and at air liquid interface for another 7 days. The metal ring was removed and the epidermis allowed to migrate outwards onto the bare DED, and the viable area of epidermis was measured by submerging in Resazurin blue stain at 50μg/ml for 1 hour at 0 and 10 days. Peptides at 200nM were added daily to the top of the skin in 2% methylcellulose (Sigma-Aldrich, Gillingham, UK) in serum-free media. Images were analysed for percentage coverage of the original wound area using ImageJ [12]. To measure the effects of peptides on the HaCaT keratinocyte cell line, a scratch assay was performed. Cells were plated in DMEM plus 10% foetal calf serum at a density of 3x10^5^ cells per well in a 24-well plate previously coated in Collagen Coating Matrix (ThermoFisher, Altrincham, UK) according to the manufacturer’s instructions. After incubation for 16 hours, a scratch was generated in the monolayer using a pipette tip and the wells were washed with PBS before treatment with 200nM peptides, or vehicle control (diluted in DMEM plus 10% serum) or in serum-free medium. Images were captured at 0 and 18 hours and the percentage coverage of the original wound was determined using ImageJ.

### Cytokine Assays

Cytokines in TESkin wound effluents were measured using cytometric bead arrays according to the protocol provided by the manufacturer (BD Bio-Science, Oxford, UK).

### Immunohistochemistry and immunofluorescence

For the determination of CD9 expression in TESkin, wax was removed from processed samples using xylene, and the tissue was rehydrated with alcohol and distilled water. Sections were then blocked with 1% BSA for 1 hour, washed with PBS and incubated for 2 hours with primary antibodies or isotype control antibody. Sections were mounted using Vectashield with DAPI (Vector Laboratories, Peterborough, UK). For the determination of tetraspanin expression on cell lines, cells were processed in 96-well plates at 1 x 10^5^ cells/well. 10μg/ml primary antibody, diluted in PBS containing 0.1% BSA and 0.2% NaN_3_, was incubated with cells on ice for 1 hour before extensive washing and incubation with the appropriate secondary antibody for 1 hour. Immunofluorescence was measured on an Applied Biosystems Attune acoustic focusing cytometer and expressed as median fluorescence intensity (MFI). A correlation between CD9 expression and the anti-adhesive effects of Antibodies used were: anti-CD9 mouse monoclonal IgG (602.29 DSHB, Iowa, USA), anti-CD151 mouse monoclonal IgG1 (14A2, kindly provided by Leonie Ashman, University of Newcastle, Australia); anti-CD81 mouse monoclonal IgG1 (1D6, AbD Serotec, Kidlington, UK), anti-CD63 mouse monoclonal IgG1 (H5C6, DSHB, Iowa, USA) mouse monoclonal IgG1 isotype control (JC1, produced in-house), rabbit anti-mouse Ig FITC conjugate (Sigma-Aldrich, Gillingham, UK), anti-staphylococcus rabbit polyclonal (Ab20920, Abcam, Cambridge, UK) and anti-rabbit Ig FITC conjugate and Alexafluor® 488 conjugated goat anti-rabbit Ig (Thermo Fisher, Rugby, UK).

#### Statistical Tests

GraphPad Prism 6 was used for all statistical analysis. Data were initially tested for skew and if this was found to be more than double the standard error, then data were considered to be non-parametrically distributed. Unless stated otherwise, data were analysed using One-Way ANOVA with Sidak’s multiple comparison post-test.

## Results

### Expression of Tetraspanins on Skin Cells

The expression of 4 common tetraspanins, CD9, CD63, CD81 and CD151, were assessed by flow cytometry (Fig 1A–1D). Both of these techniques revealed that CD9 is highly expressed on differentiated and undifferentiated primary keratinocytes and the HaCaT keratinocyte cell line, but not on dermal fibroblasts. Conversely, CD63 and CD151 are highly expressed on fibroblasts, but not on the other cell types. HaCaT cells showed expression patterns similar to those of human keratinocytes, indicating that these cells are a good model for studying tetraspanin function in human keratinocytes. CD81 is expressed highly on all cell types tested, but CD9 was chosen for further study due to its high expression levels in keratinocytes, and previous evidence of involvement in bacterial adhesion [9].

A large difference in tetraspanin expression was also observed between keratinocytes from the same donor in their proliferative state (grown in low calcium medium), and in their non-proliferative, differentiated state (Fig 1A). This suggests that CD9 is likely to be reduced in the upper more differentiated barrier layers of skin but relatively high in the lower proliferative layers of keratinocytes.

### Tetraspanin Peptides Can Reduce the Adherence of *Staphylococcus Aureus* to Host Cells

**Fig 2:**
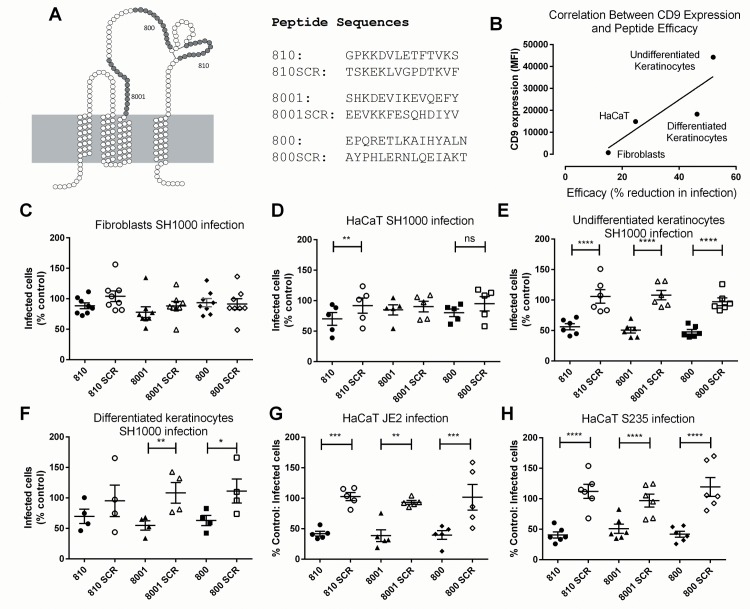
Tetraspanin Peptides Reduce *Staphylococcus Aureus* Adherence to Host Cells. (A) Peptide sequences and their origin in CD9 extracellular 2 domain. (B) Correlation between maximal peptide efficacy on SH1000 adhesion and CD9 expression. (C-H) Cells were treated with 50nM peptide or scrambled control for 30 minutes, then infected with bacteria for 1 hour. They were then fixed and bacteria quantified by fluorescence microscopy. Data are presented as % of an untreated control, transformed by Y = log_10_(Y) before analysis with one-way ANOVA. * p≤0.05 **p≤0.01 ***p≤0.001 ***p˂0.0001.

CD9 reagents (anti-tetraspanin antibodies and recombinant tetraspanin large extracellular domains) have previously been shown to reduce the adherence of various strains of both Gram negative and Gram positive bacteria to human epithelial cell lines [13]. In this study we tested the efficacy of 3 short peptides, 810, 8001 and 800, derived from the primary sequence of the tetraspanin CD9 large extracellular domain (Fig 2A), on *Staphylococcus aureus* binding to human keratinocyte cells; scrambled peptides (SCR) consisting of the same amino acids in a random order were used as controls.

Human keratinocyte-like HaCaT cells or primary human keratinocytes were pre-treated with 50nM peptide (or scrambled controls) then infected with 3 well-characterised *S*. *aureus* strains of increasing virulence: SH1000, S235 or JE2 MRSA (Fig 2C–2H, raw data in S1 Fig). When HaCaT cells were infected with S235 and JE2 clinical isolate strains of *S*. *aureus*, significant reductions in bacterial adherence were observed with all three tetraspanin-derived peptides. HaCaT cells pre-treated with peptide 810 had a maximum reduction of adherence of S235 strain of 60% compared to its scrambled control, and a maximum reduction of 56% was seen with 800 peptide on JE2 bacterial adherence. The anti-adherence effect was also observed in primary keratinocytes isolated from multiple donors following infection with the laboratory-adapted low virulence strain SH1000, with a significant reduction of approximately 52% with peptide 800. Interestingly, the adherence of SH1000 to HaCaT cells was only significantly inhibited by peptide 810 and there was no effect of peptide treatment on SH1000 attachment to primary human skin fibroblasts. The expression level of CD9 on each cell type showed a significant correlation (R^2^ = 0.74; slope significantly non-zero, p < 0.01) with the efficacy of the most effective peptide treatment in reducing bacterial attachment (Fig 2B).

### Fluorescently-Tagged Peptide 800 Interacts with Primary Keratinocytes

**Fig 3:**
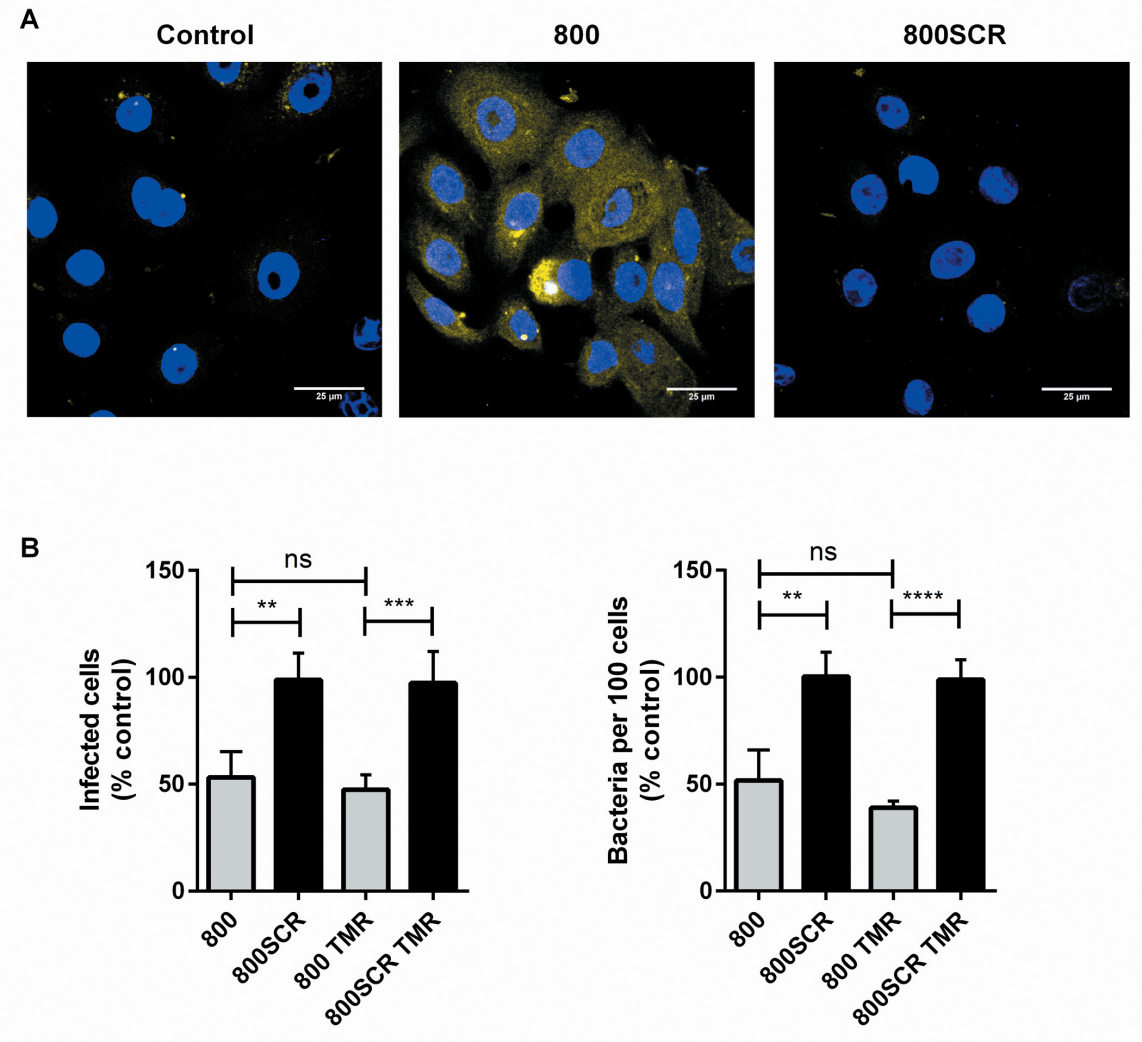
Peptides Interact with Keratinocytes. (A) 800 peptide or 800SCR tagged with tetramethylrhodamine (TMR) were added to primary keratinocytes at a concentration of 200nM for 30 minutes, then fixed and viewed by confocal microscopy. Scale bars: 25μM. (B) Cells were treated with no peptide (control), 50nM untagged peptide 800, the TMR-tagged form or its scrambled peptide for 30 minutes, then infected with bacteria for 1 hour. They were then fixed and bacteria quantified by fluorescence microscopy. Data are presented as % of an untreated control, transformed by Y = log_10_(Y) before analysis with one-way ANOVA. **p≤0.01 ***p˂0.0001.

In order to provide some insight into the mechanism of action of the tetraspanin peptides, we examined the interaction of tetramethylrhodamine (TMR)-tagged peptides 800 and 800SCR with primary keratinocytes adhered to glass coverslips. Peptide 800 but not the scrambled control peptide is bound by the keratinocytes (Fig 3A). The membrane staining visible here supports the hypothesis that the peptides interact with host membranes as opposed to the bacteria. The TMR-tagged 800 peptide was still functionally active and capable of reducing the adherence of *S*. *aureus* to host cells whereas its scrambled control form was not (Fig 3B).

### The Effects of Peptide 800 Are Time- and Dose-Dependent

**Fig 4:**
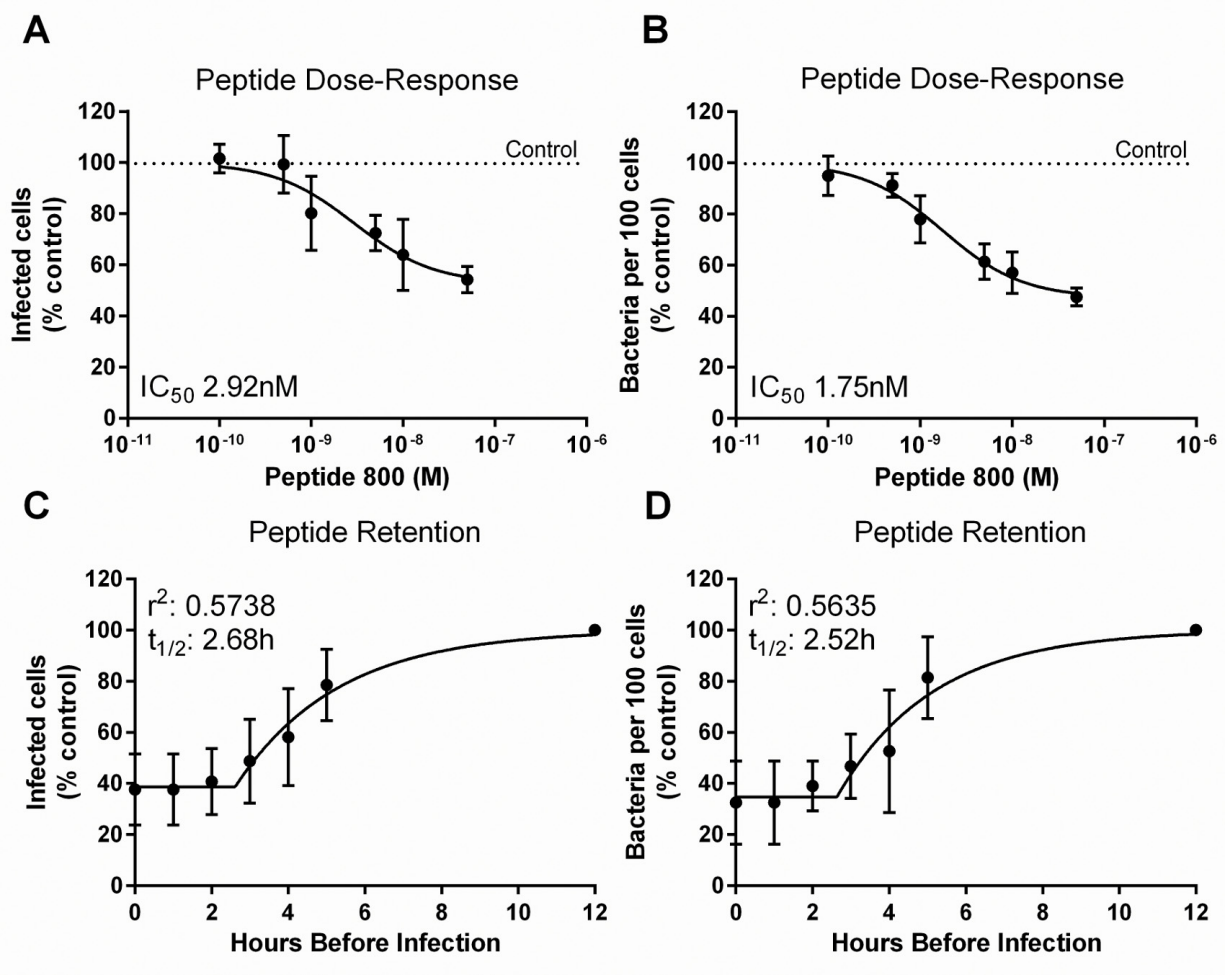
Dose- and Time-Dependent Effects of Tetraspanin Peptide. (A and B) Varying dosages of 800 peptide were applied to HaCaT cells for 30 minutes then infected with bacteria S235 strain *S*. *aureus* for 1 hour before fixing and analysis. (C and D) 50nM peptide was applied to HaCaT cells at various time points then washed away after 1 hour. S235 Bacteria were then added at an MOI of 30 for one hour. Best fit was obtained when data were modelled using plateau followed by one-phase dissociation. A and C represent the proportion of infected cells, B and D represent the number of bacteria per 100 cells.

A dose response experiment was carried out to ascertain the IC_50_ for 800 peptide (Fig 4A and 4B) on Staphylococcal (S235 strain) adherence. HaCaT cells were pre-treated for 30 minutes with different concentrations of peptide then infected for 1 hour before fixation and quantification. The results indicate that the peptides retain inhibitory function at concentrations as low as 1nM, with an IC_50_ between 1.8 and 2.9 nM, but the effect was lost completely at concentrations below 0.5nM. To determine the persistence of the effect of 800 peptide, HaCaT cells were treated with 800 peptide, then washed and cultured in media for 0–5 hours before being infected with S235 strain for 1 hour (Fig 4C and 4D). The data show that the peptide is fully effective at reducing the adherence of bacteria to cells for up to 2.5 hours. This effect begins to diminish with a t_1/2_ of 2.52/2.68h, however the effect is not completely lost until approximately 8 hours after wash-out.

### CD9 Is Expressed In a 3D Tissue Engineered Model of Human Skin

**Fig 5:**
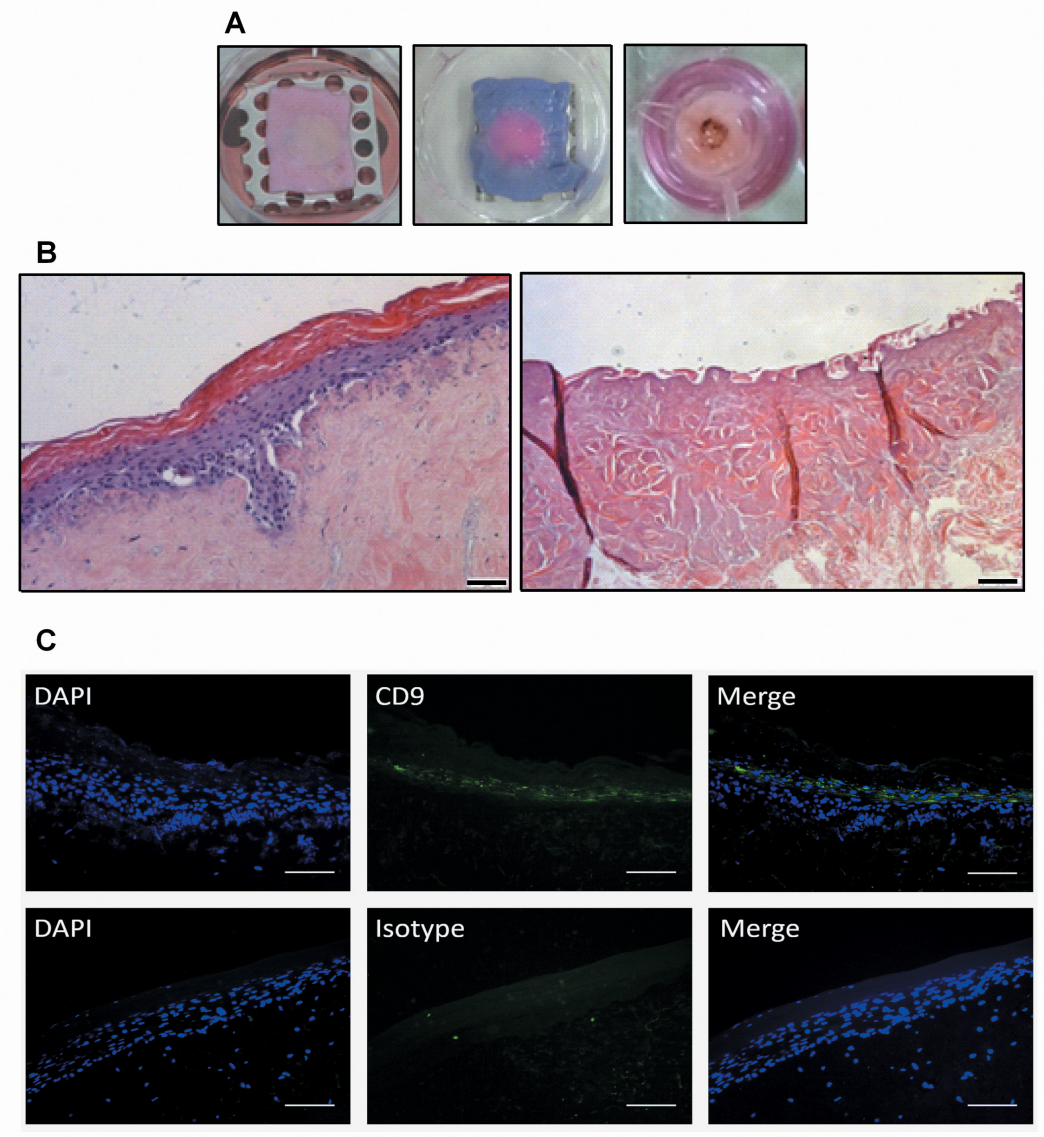
Tissue Engineered Model of Human Skin. TEskin was constructed as described in Methods and Materials and grown at an air-liquid interface for 14 days. (A) TEskin in which the cells have been seeded and grown in the centre of a metal ring (left panel). A Resazurin blue stain highlights the metabolically active area in pink (middle panel). To generate a wound, the TEskin was burnt in the centre with a heated metal rod (right panel. (B) H&E staining of unwounded (left panel) and wounded (right panel) TEskin. Scale bars = 75μm (C) CD9 immunohistochemistry staining reveals high CD9 expression in the spinous and granular layers of the epidermis. Scale bars = 100μm.

Although cell lines are a good preliminary method for determining the effects of the tetraspanin-based treatments in a simple system, they provide only limited information about how the therapy would work in a more complex tissue such as skin. To this end, 3D tissue engineered skin (TEskin) [10], was used to model *Staphylococcus aureus* infected wounds in human skin (Fig 5A). TEskin mimics the tissue structure of normal adult human skin and can be used to analyse the penetration of peptides and bacteria, and the effectiveness of the peptides in a more relevant model. Fig 5B shows a cross section of TEskin after 14 days growth at air-liquid interface (ALI) stained with haematoxylin and eosin. The cells migrate and differentiate within the de-epidermised acellular dermal scaffold (DED) to form a fibroblast-seeded dermis, an epidermis consisting of granular, basal and spinous keratinocytes, and a fully stratified stratum corneum. When the model is wounded, much of the epidermis is lost and the dermis is exposed. At the periphery of the wounded area, lower levels of the epidermis are exposed leaving undifferentiated keratinocytes susceptible to infection.

Sections of TEskin were stained for CD9 expression, and this was identified primarily in the lower levels of the epidermis (Fig 5C). No CD9 was observed in the upper layers, where the cells would be expected to be the most differentiated. This result is consistent with our observations from monolayer cultured keratinocytes.

### Pre-Treatment with Peptide 800 Decreases the Bacterial Burden in a TEskin Wound Model

**Fig 6:**
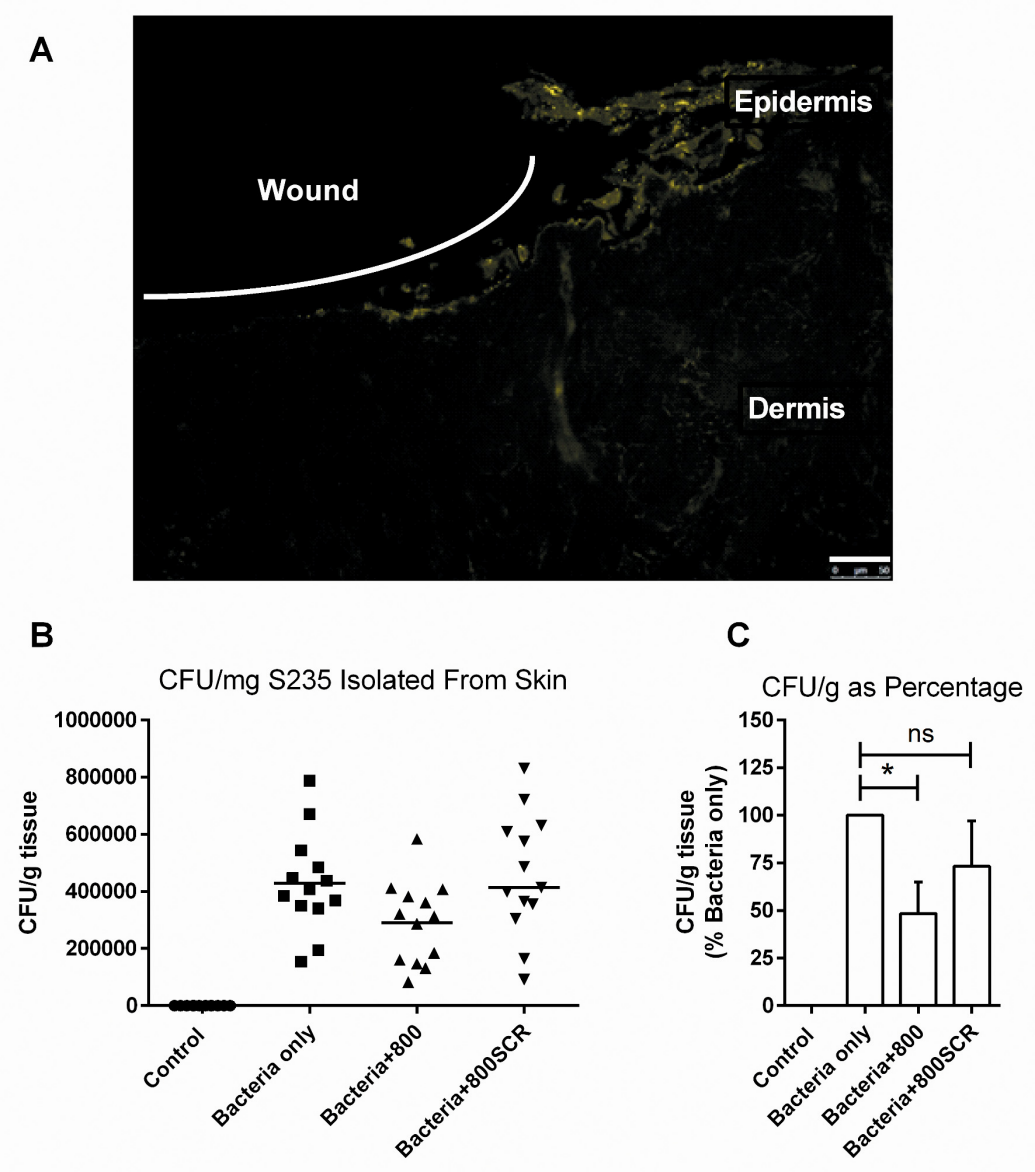
Peptides Reduce Adherence to TEskin. (A) Wounded TEskin infected with S235 strain bacteria was stained using anti-staphylococcal rabbit polyclonal antibody and anti-rabbit immunoglobulins Alexafluor® 488 conjugated goat secondary shown here in yellow. (B and C) TEskin was pre-treated with 500nM 800 or 800SCR peptide then infected with S235 bacteria over 24 hours. * p≤0.05. n = 4, performed in triplicate. Scale bars = 50μm.

After 1 hour of peptide or control treatment, 1 x 10^7^ S235 bacteria were added to the top of the wounded TEskin and allowed to adhere for 5 hours. The TEskin was then washed to remove non-adherent bacteria, and the remaining pathogen allowed to grow and infect until 24 hours after wounding, when they were washed again. The TEskin was then fixed, and bacteria visualised by indirect antibody staining and the nuclei by DAPI staining (Fig 6A). Even after 24 hours, infecting bacteria were localised to the surface of the skin, with some infecting the remaining epidermis, but very few penetrating deeper into the dermis. TEskin in these conditions was also treated with saponin in order to isolate the bacteria for viable cell counts to identify any effects of peptide treatment (Fig 6B and 6C). Treatment with 800 peptide reduced the quantity of adherent bacteria to around 52% of the untreated control value. This reduction in bacteria indicate a less severe infection in the TEskin treated with peptide, suggesting that the peptides might be interesting candidates for the development of anti-adherence therapy. This reduction in infection was not seen with the 800SCR peptide.

### Tetraspanin-Derived Peptides Do Not Affect Keratinocyte Viability, Epidermal Migration or Bacterial Viability

**Fig 7:**
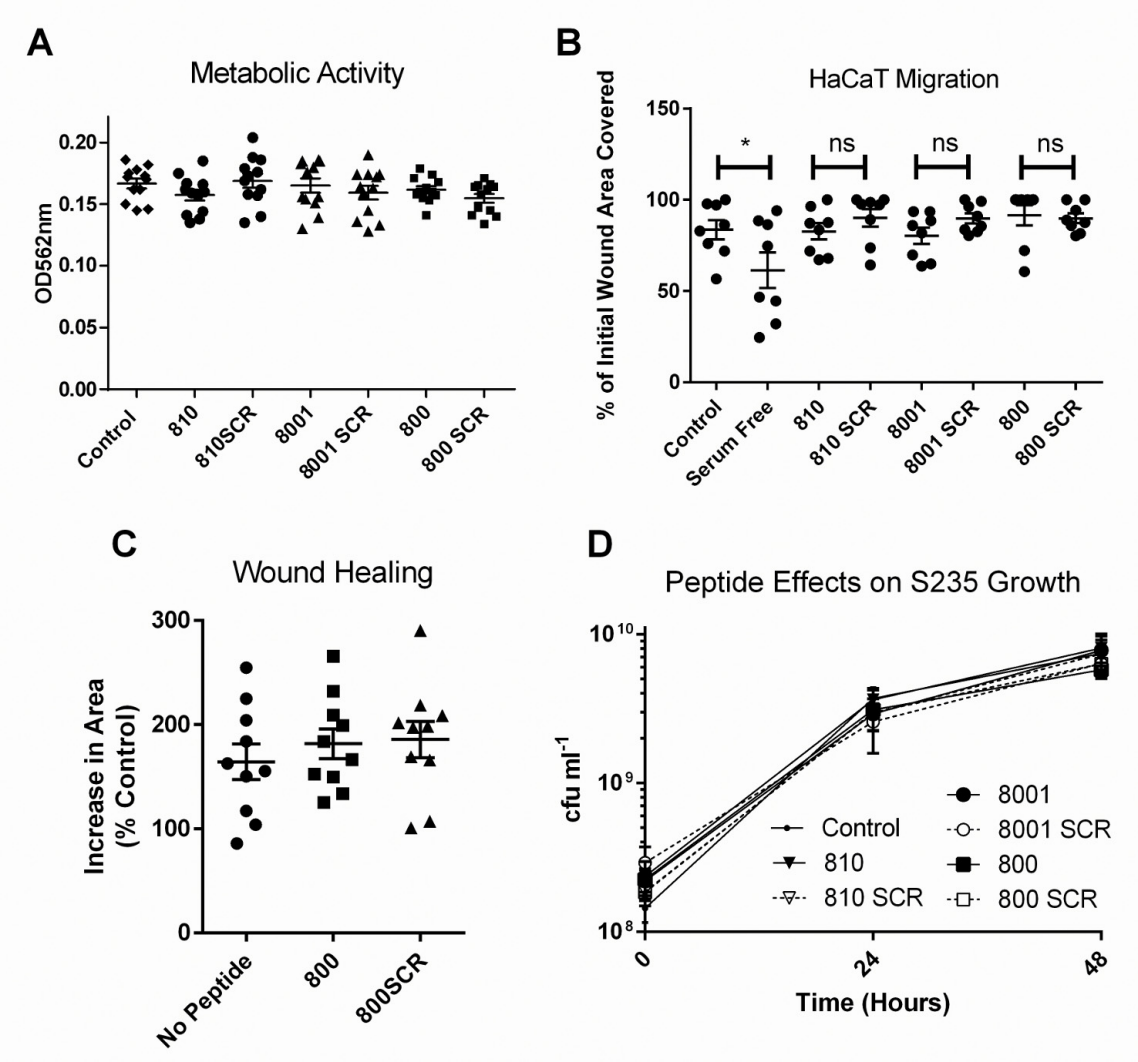
Tetraspanin Peptides Do Not Affect Bacterial Viability of Cell Migration. (A) HaCaT cells were seeded into a 24 well plate then treated with MTT stain for 1 hour. They were then permeabilised and the supernatant analysed using a plate reader at 562nm n = 3, duplicate. (B) HaCat cells were seeded into a 24 well plate to form a confluent monolayer. A scratch was made using a pipette tip, and the healing rate quantified using ImageJ, n = 5, performed in duplicate, Analysis by one-way ANOVA, ****p˂0.0001. (C) A TEskin model was set up to measure the migration of the epidermis across bare dermis over 10 days, with constant peptide treatment. Measurements were taken at 0 and 10 days using a Resazurin blue stain and ImageJ area measurements tools, n = 3 (D) S235 Bacteria were grown for 48 hours in the presence of 200nM peptide, and CFU measurements taken. n = 3, performed in duplicate.

The peptides were also tested for any negative effects on skin cell viability and function. None of the peptides had any effect on keratinocyte cell metabolism when compared to their scrambled controls at 200nM or when compared to a media alone using an MTT cell viability Assay (Fig 7A). However, there was a small, non-significant reduction in the healing rate of cells treated with 800 peptide as quantified by a scratch assay (Fig 7B). To determine if 800 peptide could affect the migration and proliferation essential for wound healing, the TEskin model was constructed with the keratinocytes and fibroblasts grown onto DED but contained in the centre of a metal ring until fully formed and stratified. This ring was removed after 7 days at air liquid interface, and the live keratinocytes and fibroblasts allowed to re-epithelise the bare dermis, representative of the exposed dermis of a wound. The area of viable cells was measured by Resazurin Blue staining and ImageJ analysis at 0 and 10 days after removal of the metal ring (Fig 7C). TEskin was treated with 200nM peptides every two days throughout the experiment. No negative effects on the essential wound healing process were observed in this more complex model at any of the time points observed, and so 800 peptide was deemed to not have a negative effect on cell migration and proliferation. Additionally, no direct effects on the growth and viability of S235 strain *Staphylococcus aureus* (Fig 7D) were seen with 200nM peptide treatment.

### Tetraspanin Peptides Do Not Affect Cytokine Production in TEskin

Tetraspanins are involved in cell signalling and immunity, and so the possibility that the peptides could affect immune functions of keratinocytes was tested using a cytometric bead array (CBA) to detect any changes in cytokine or chemokine production in TEskin. Samples were collected 24 hours post wounding, in serum free media. In a preliminary experiment, a small number of samples were screened for the cytokines known to be secreted by keratinocytes and fibroblasts, to assess which could also be detected in wound effluent from TEskin. Detectable levels of interleukins IL-6, IL-8, CCL2 (MCP-1) and IL-1α were produced in TEskin.

**Fig 8:**
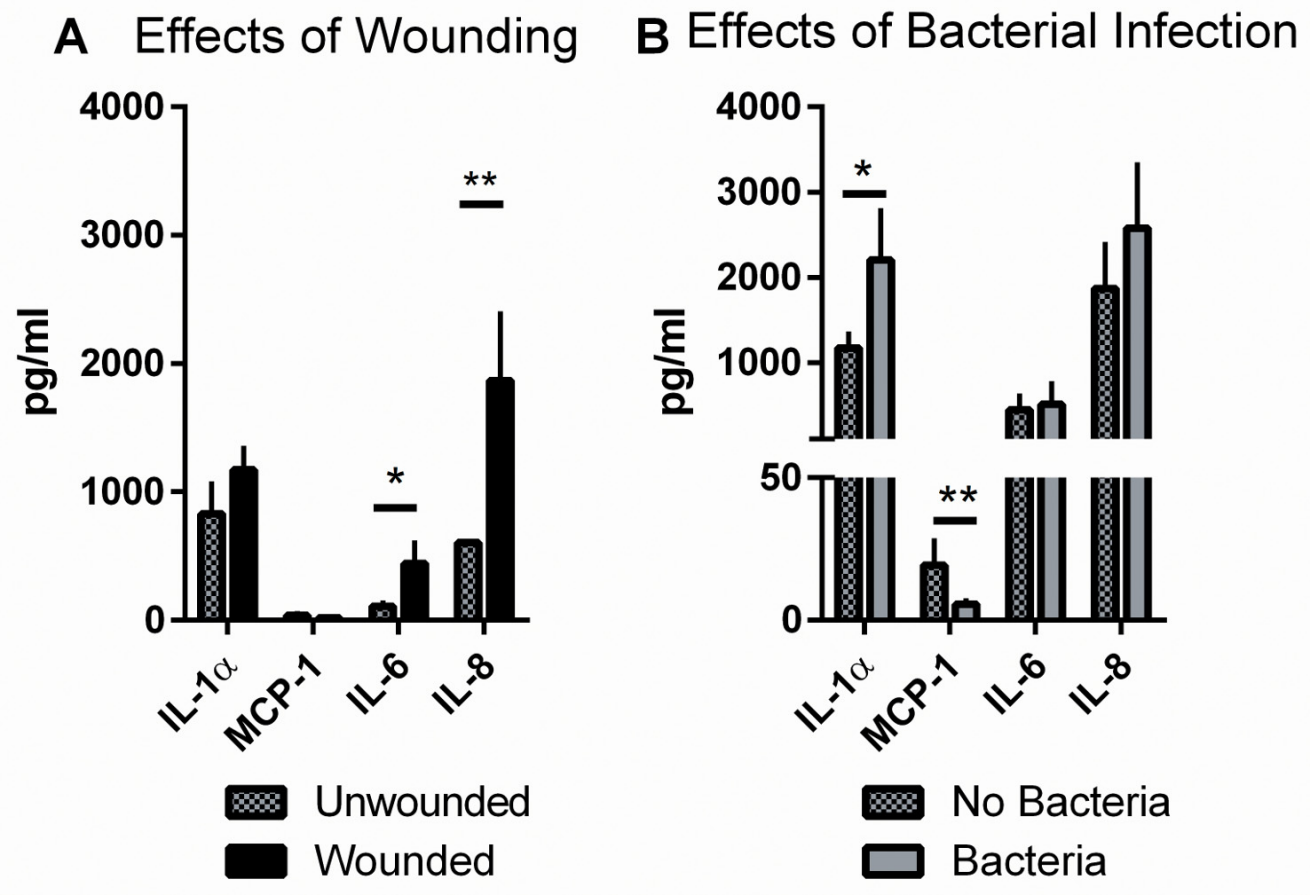
Wounding and Infection Affect Cytokine Production by TEskin. (A) TEskin was wounded by burning, or left intact. (B) TEskin was burned, and either infected with S235 strain Staphylococcus aureus or left uninfected. Cytokines were taken at 24 hours, and measured using a cytometric bead array. Data analysed by unpaired T test, * p≤0.05 **p≤0.01. n = 3, performed in triplicate.

**Fig 9:**
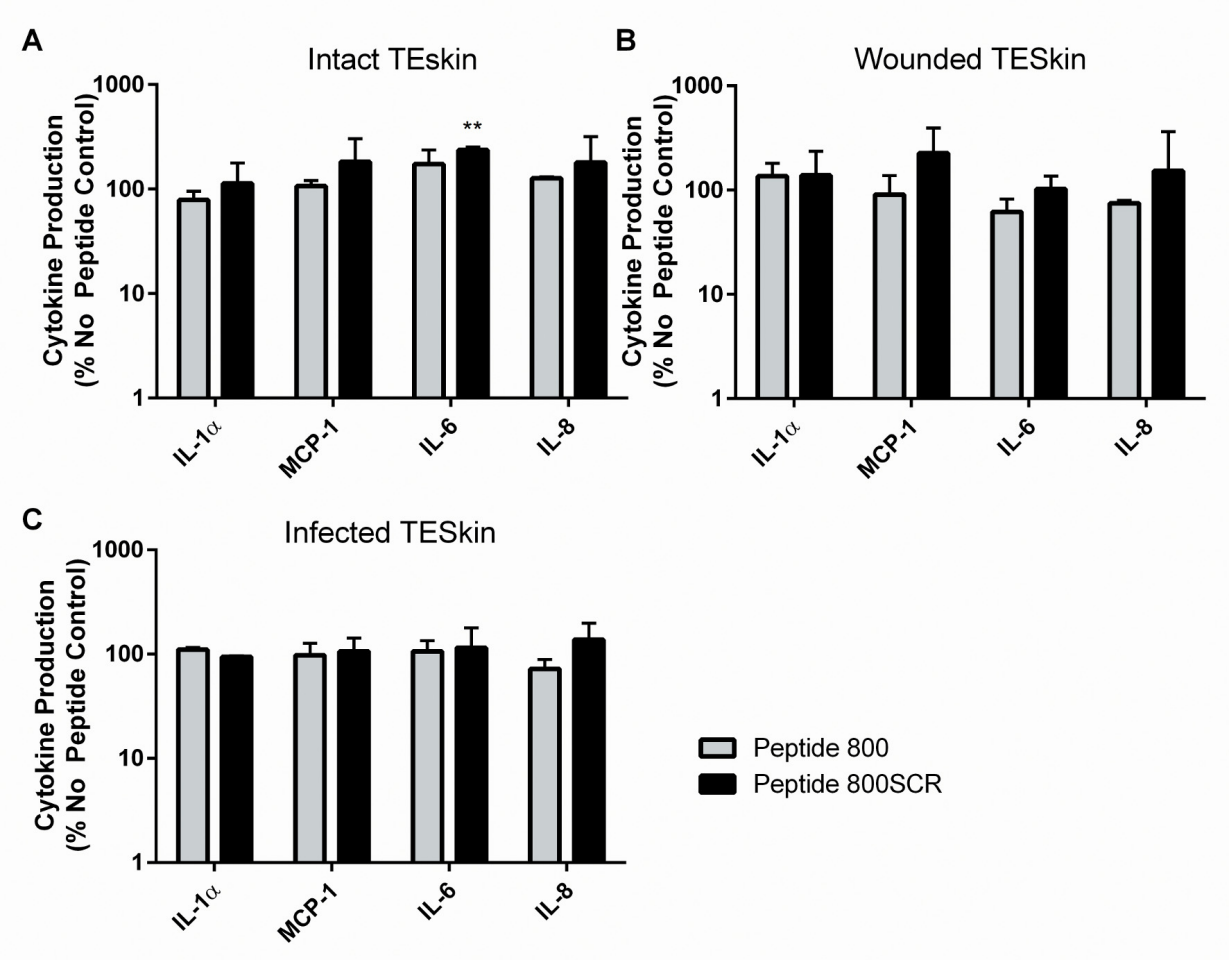
Tetraspanin Peptides Do Not Affect Cytokine Production by TEskin. TEskin in various states was treated with peptide 800 or 800SCR at 200nM, or serum free media as a control. Production of IL-1α, MCP-1, IL-6 and IL-8 were measured at 24 hours post wounding/treatment using cytometric bead array. (A) Unwounded, uninfected (B) Wounded, uninfected (C) Wounded, infected. Data transformed by Y = log_10_Y and analysed by One-way ANOVA**p≤0.01 n = 3, performed in triplicate.

Wound effluent was collected at 24 hours post wounding/treatment from TEskin in various conditions: wounded and mock-wounded, infected or sterile, and with or without peptide treatment. Fig 8 shows that differences in cytokine levels occur in response to wounding and to wound infection by S235 strain *Staphylococcus aureus*. 24 hours after burning TEskin, a significant increase in the levels of IL-6 and IL-8 was detected compared to the levels secreted by intact, unwounded TEskin. A small but non-significant increase in IL-1α levels was also seen. When burned TEskin was infected with S235 bacteria, a significant increase in IL-1α was observed after 24 hours, along with a significant reduction in chemokine CCL2. No change was observed with IL-6 or IL-8 (Fig 8B). To assess if treatment with 800 peptide had any effect on the cytokine response, assays were performed with 200nM peptide added to the skin throughout the 24 hour incubation (Fig 9A–9C). No effect was seen on cytokine secretion by TEskin with 200nM 800 peptide treatment. There was a small but significant increase in the secretion of IL-6 with peptide 800SCR, perhaps due to a contaminant in the peptide preparation or a non-specific effect of the scrambled sequence.

## Discussion

Here we have shown that peptides based on the EC2 domain of the tetraspanin CD9 can reduce the adherence of various MSSA and MRSA strains to skin cells in monolayer cultures, and to a 3D model of human skin. We also observed no adverse effects of the peptides on cell metabolism and migration.

The expression level of CD9 on each cell type correlated weakly with the efficacy of the peptide treatment on bacterial attachment and so one explanation for the lack of sensitivity of skin fibroblasts to the CD9-derived peptides may be the very low CD9 expression by these cells, in contrast to high levels of expression of CD9 expressed by both primary keratinocytes and the HaCaT line. CD9 expression falls when primary keratinocytes are differentiated into a more mature keratinocyte phenotype by culture in high calcium medium, and differentiated keratinocytes bind relatively low numbers of bacteria, suggesting that a different binding mechanism may be responsible for bacterial adherence to CD9-low keratinocytes. As only approximately 50–60% of bacterial adherence is affected by peptide treatment, even at very high concentrations of peptide, the remaining attachment could be due to a variety of CD9-independent mechanisms, which might also explain the differences in peptide efficacy on different staphylococcal strains. The adherence of bacteria to fibroblasts appears also to be independent of CD9 expression, and so it would be interesting to test peptides from other tetraspanins to determine if the mechanism is completely tetraspanin-independent.

Tetraspanin peptides are active on the host cell rather than on the bacteria themselves, as they have no toxicity to the bacteria and have no anti-adherence activity when added to the bacteria before adhesion to host cells. Although we provide no evidence of a mechanism for the peptides here, other than to demonstrate a specific interaction of the active form of the peptide with human keratinocytes, it is likely that peptides are acting in a similar manner to recombinant CD9 EC2 protein which has been shown to inhibit the interaction between lymphocytes and endothelial cells by decreasing the clustering of TEM-associated adhesion proteins such as ICAM-1 [14]. Because of this, it was important to test for any interference with normal cellular function by the peptides. Specifically, a role for tetraspanins in cell migration and wound healing has been observed [15], although later studies found that blocking CD9 with antibodies actually increased HaCaT migration in a cell scratch assay [16]. In this study, peptides had no significant effects on the migration speed of HaCaTs or of keratinocytes in an epidermis. This may be because the peptides are much smaller than antibodies and so less capable of sterically hindering the formation of motility-related structures, or because the effects were not large enough to be observed in these systems. Tetraspanins are also known to be involved with immune responses and so we looked at cytokine secretion by the peptides and saw no significant differences with our active peptide 800. The TEskin model used however is only composed of fibroblasts and keratinocytes and there are no immune cells, and therefore we cannot comment on the effects of the peptides on the immune system. It would be interesting to test for the effects of peptides and EC2 domains on various immune functions such as dendritic cell function and cell signalling.

Anti-adherence therapies are a relatively new area of research [17]. Most commercially available antimicrobials work by directly killing or inhibiting the growth of the pathogen and so selective pressure can lead rapidly to the emergence of resistant strains such as MRSA. Unlike traditional drugs, tetraspanin based anti-adherence peptides target components of the host as opposed to the bacteria and thus have a decreased likelihood of the development of resistance [18]. This, alongside their broad specificity and low toxicity, make tetraspanin-derived peptides an appealing candidate for anti-bacterial drug development.

## Supporting Information

S1 Fig Raw Adherence Data. Raw data from cell line infection experiments, conducted in duplicate. (A) Fibroblasts and SH1000, MOI 5, n = 8 (B) HaCaTs and SH1000, MOI 160, n = 6 (C) Differentiated keratinocytes and SH1000, MOI 30, n = 4 (D) Undifferentiated keratinocytes and SH1000, MOI 30, n = 6 (E) HaCaTs and JE2, MOI 200, n = 6 (F) HaCaTs and JE2, MOI 40, n = 6. (G, H) show the effect of tetramethylrhodamine (TMR) labelled peptide 800 on SH1000 infection of HaCaT cells (MOI 30), n = 6. Percentage data transformed by Y = log_10_Y before analysis by one-way ANOVA with Sidak’s multiple comparisons.
(TIF)
